# Protein A chromatography increases monoclonal antibody aggregation rate during subsequent low pH virus inactivation hold

**DOI:** 10.1016/j.chroma.2015.08.068

**Published:** 2015-10-09

**Authors:** Alice R. Mazzer, Xavier Perraud, Jennifer Halley, John O’Hara, Daniel G. Bracewell

**Affiliations:** a Department of Biochemical Engineering, University College London, Bernard Katz Building, Gordon Street, London WC1H 0AH, United Kingdom; b UCB Celltech, 216 Bath Road, Slough SL13WE, United Kingdom

**Keywords:** Aggregation, IgG, Size exclusion chromatography, Affinity adsorption, Unfolding pH

## Abstract

Protein A chromatography is a near-ubiquitous method of mAb capture in bioprocesses. The use of low pH buffer for elution from protein A is known to contribute to product aggregation. Yet, a more limited set of evidence suggests that low pH may not be the sole cause of aggregation in protein A chromatography, rather, other facets of the process may contribute significantly. This paper presents a well-defined method for investigating this problem. An IgG4 was incubated in elution buffer after protein A chromatography (typical of the viral inactivation hold) and the quantity of monomer in neutralised samples was determined by size exclusion chromatography; elution buffers of different pH values predetermined to induce aggregation of the IgG4 were used. Rate constants for monomer decay over time were determined by fitting exponential decay functions to the data. Similar experiments were implemented in the absence of a chromatography step, i.e. IgG4 aggregation at low pH. Rate constants for aggregation after protein A chromatography were considerably higher than those from low pH exposure alone; a distinct shift in aggregation rates was apparent across the pH range tested.

## Highlights

• IgG aggregation at low pH follows pH-dependent exponential decay kinetics.
• IgG aggregation behaviour is influenced by a protein A chromatography step.
• The mechanism of aggregation appears to be accelerated by the separation process.

## 1 Introduction

Therapeutic monoclonal antibodies (mAbs) occupy a significant portion of the biopharmaceutical market; 29 new mAbs are presently undergoing late-stage clinical trials, including human and humanised IgG1, 2 and 4 molecules, amongst others [1]. Industrial-scale production of biopharmaceuticals is a challenging task; preserving the product by limiting stresses that may cause degradation, combined with maximising yield and minimising resources consumed are key elements of bioprocess development. One form of protein degradation is aggregation. In the biopharmaceutical industry, aggregates are classed as impurities and must be cleared to acceptable levels if a protein formulation is to be used therapeutically. Aggregates are thought to pose a risk of unwanted immunogenicity, and may affect product potency and reproducibility [2–5].

After the initial product recovery stages of a bioprocess, the “capture” stage takes place, whereby substantial purification and concentration of the product is effected in a single step [6]. A near-ubiquitous method of IgG capture is protein A affinity chromatography. *Staphylococcal* protein A binds all IgG molecules of subclasses 1, 2 and 4 [7] with high selectivity and minimal interaction with the Fab region [8], the active region of the drug molecule. Product molecules are eluted from protein A resins by lowering the pH; a typical elution buffer is 0.1 M sodium citrate, pH 3.3. Low pH is often maintained for a period of time for the purpose of viral inactivation [9,10]. However, for many antibodies, acidic conditions and sudden pH changes can result in aggregation [3,7,9–11].

Protein aggregation induced by pH has been the subject of much investigation in bioprocess development, and low pH is typically cited as the cause of product aggregation occurring during or after protein A [12] chromatography; it is also acknowledged that protein aggregation often occurs more readily at high concentrations. Further to this, a more limited pool of evidence suggests that low pH may not be the sole cause of aggregation in protein A chromatography, rather, the adsorption and desorption events themselves may contribute significantly [6,9,13].

A typical model for protein aggregation consists of four stages: reversible destabilisation of native structure or partial unfolding to form the “reactive monomer” (*R*_*M*_) [14]; reversible or irreversible association of *R*_*M*_ yielding a more thermodynamically favourable “aggregate” state; association of a critical number of *R*_*M*_ to form a nucleus; addition of *R*_*M*_ or small oligomers to the nucleus to form larger amorphous or ordered aggregates [14]. Different theories argue different stages as the rate limiting step [2,14]. For the first stage, the destabilising effect of low pH on IgG4 has been shown using differential scanning calorimetry (DSC), a method for assessing thermal unfolding. At pH 6.0, two major endothermic transitions are seen; at pH 3.5 similar transitions are seen at significantly lower temperatures; at pH 2.7 a very different transition profile is seen, with major transitions occurring at relatively low temperatures [10].

A study by Shukla et al. attempted to elucidate the collective effects of low pH, protein concentration and chromatographic separation on an Fc-fusion protein [9]. Shukla et al. found that protein A chromatography significantly increased the rate constant for formation of high molecular weight species at low pH. Rate constants were determined graphically using a derivation of a Lumry–Eyring-based kinetic model for monomer loss/aggregate formation. In this instance rate constants were found to be concentration-independent. Different additives included in the chromatography elution buffer significantly altered aggregation rates. Interestingly, in some cases, additives that stabilised proteins at low pH in solution had destabilising effects in chromatography experiments. Urea was an effective additive in reducing on-column and in-solution aggregation at concentrations of 0.5 M and 1 M, respectively [9]. In other work, arginine was found to prevent protein aggregation on elution from protein A [6]. Recent work by Gagnon et al. showed that elution from protein A affects the conformation of IgG1; namely, a significantly reduced hydrodynamic radius and changes in secondary structure content were observed. Notably these effects did not occur at low pH in the absence of the elution event [13].

Protein A binds to the Fc region of IgG about the hinge between the C_H_2 and C_H_3 domains; it contains five highly-homologous Fc-binding domains and can bind at least two IgG molecules simultaneously [15–18]. The precise region of the Fc that binds to protein A can also bind a number of other molecules; thus, it has been termed *consensus binding site* (CBS) [19]. The CBS is largely hydrophobic in character, contains relatively few polar residues and has a high level of solvent accessibility. These features indicate burial of hydrophobic residues as a strong driver of binding [19,20]. Electrostatic interactions [8] and hydrogen bonds at certain highly conserved sites [21] have also been indicated in binding. According to Delano et al., the CBS undergoes considerable conformational changes when binding to a ligand. In fact, the nature of the change in conformation depends on the ligand, highlighting the flexibility of this region [19]. Though flexibility implies good structural recovery after conformational change, under antagonistic conditions such as low pH there may be greater vulnerability to detrimental levels of structural alteration. X-ray crystallography studies by Deisenhofer found that C_H_2 domain disorder was greater in an Fc-Fragment B complex (Fragment B being a single protein A domain) than in the unbound Fc, implying potential Fc destabilisation on adsorption to protein A [13,20]. However, this may not have been the case had the Fab fragment been present, due to C_H_2-Fab contact [20]. Conversely, it has been shown that some IgG molecules retain a folded structure at pH conditions as low as pH 2.7 after elution from a protein A column, though it is not identical to the native structure. In this case, IgG aggregation after low pH elution was instead attributed to conformational disturbances occurring during neutralisation of low pH samples [10].

Predictably, the literature indicates great variation in protein aggregation behaviour depending on the protein. Though aggregation in protein A elution pools is well-acknowledged, its mechanism and impact is not well-understood. The aim of this work is to clearly present a method showing that the process of protein A chromatography increases the rate of aggregation of an IgG4 molecule at low pH. Experiments are designed to mimic the part of a standard mAb bioprocess where protein A chromatography is followed by incubation at low pH for the purpose of virus inactivation, and subsequent neutralisation of this pool. MabSelect Xtra (GE Healthcare), which consists of agarose particles with an engineered recombinant protein A ligand, will be used as this is a modern industrially relevant resin. Importance is placed on production of reliable kinetic data and strong comparability between data sets where a chromatography step is included, and those done in solution only at low pH.

## 2 Materials and methods

### 2.1 Materials

The IgG molecule used for all experiments was kindly donated by UCB Celltech (UCB Celltech, Slough, UK). It is a purified IgG4 kappa antibody; the hinge region is not mutated. The IgG is formulated at 17.8 mg/mL in 270 mM glycine, 1% maltose, pH 5.0. This IgG4 kappa has a P_I_ between 6.85 and 8.15.

### 2.2 Equipment

Size exclusion chromatography (SEC) was carried out using an Agilent 1100 HPLC system with ChemStation software (Agilent Technologies UK Ltd., Berkshire, UK) with a TSK Gel 7 mm × 300 mm G3000SWXL column (Tosoh Bioscience LLC, Montgomeryville, USA). Affinity chromatography was carried out using an AKTA Avant 25 liquid chromatography system with Unicorn 6 software (GE Healthcare, Uppsala, Sweden), with a 1 mL HiTrap MabSelect Xtra column (GE Healthcare UK Ltd., Buckinghamshire, UK) attached.

### 2.3 Methods

#### 2.3.1 Solution experiments

Solution experiments were carried out initially to determine an appropriate pH range to work in with this IgG4 molecule, and timescales for aggregation.

The IgG was mixed with various solutions of glycine–HCl to produce solutions of IgG at concentrations ranging from 0.9 mg/mL to 4.5 mg/mL, pH conditions ranging from pH 2.78 to pH 3.03, and a buffer concentration of 0.15 M glycine–HCl. Proportions of reagents needed to give the desired pH were calculated using the Henderson–Hasselbalch equation with an adjustment for fixed ionic strength [22]. pH was measured using a standard laboratory pH probe; pH adjustment was not implemented and reported pH values represent the *measured* values. Concentrations recorded are calculated values based on the initial concentration reported for the drug substance and the subsequent level of dilution in low pH buffer. Samples were taken from each solution at various time points and neutralised with a 10% volume of 0.8 M Tris–HCl, pH 8.45. Neutralised samples were diluted in SEC mobile phase buffer. Samples were stored at 4 °C overnight before analysis by SEC. All conditions were run in duplicate. Controls consisted of IgG4 diluted to various concentrations in SEC buffer and run on the SEC column. The order of analysis of all samples (including controls) by SEC was randomised. Peaks were detected by absorbance at 280 nm. The following equation was used to determine percentage monomer recovered:

(1) $$R=\left(\frac{A_{S}}{A_{C}}\right)\times 100$$

where *R* is the percentage monomer recovered, and *A*_*S*_ and *A*_*C*_ are the sample and control monomer peak areas (mAU^2^), respectively. Percentage monomer recovered was plotted against incubation time to determine rate of monomer loss.

#### 2.3.2 Column experiments

A 1 mL HiTrap MabSelect Xtra column was equilibrated with 3 column volumes (CV) 0.02 M sodium phosphate, pH 6.7, and 2 mL of 5.6 mg/mL IgG4 was loaded onto the column using a sample loop. The column was washed with 3CV (sufficient to return baseline to zero) 0.02 M sodium phosphate, pH 6.7, and step elution of the protein was effected with 5CV 0.15 M glycine–HCl, pH 2.93. The column effluent was monitored by absorbance at 280 nm and 0.5 mL fractions were collected throughout elution. The delay volume between the detector and the fraction collector on the AKTA system was 594 μL. Protein concentration in each fraction was measured offline and showed that fraction location was consistent with the UV trace from the AKTA software. The pH was monitored using the in-line pH metre supplied with the AKTA instrument which was calibrated for the elution pH range (between pH 1.68 and pH 4.00); in-line pH measurement was used only as an indicative measure before offline measurement of individual fractions. The majority of the elution peak was collected into three central fractions. The third of these fractions, containing the tail end of the peak, was used for subsequent experimentation as this fraction was at an appropriate pH to induce aggregation. This will be referred to as *fraction* 3. Fraction 3 was mixed to ensure homogeneity and incubated at the elution pH. The pH of all elution fractions was verified at the end of the incubation using a PHR 146B Micro Combination pH Electrode (Lazar Research Laboratories, Los Angeles, CA, USA). Chromatography “dry runs” were also carried out in the absence of IgG and the pH of the elution fractions were measured individually. Fraction 3 was found to be at the pH of the original elution buffer, ±0.03 pH units. Samples of fraction 3 were neutralised at various time points, then diluted in SEC mobile phase buffer and stored overnight before SEC analysis as in Section 2.3.1. The protein concentration in these samples was determined by measuring the absorbance of each sample at 280 nm using a NanoDrop 1000 spectrophotometer (Thermo Scientific, Wilmington, DE, USA), and using Beer's law and an extinction coefficient $E_{1\;\text{cm}}^{0.1\%}=1.61$ (experimentally determined by UCB Celltech, Slough). Control samples, for calculation of percentage monomer loss, consisted of the column feed (IgG diluted in equilibration buffer), the concentration of which was also verified by UV absorbance measurement. Controls for “effect of column alone” consisted of samples from the leading side of the elution peak; on exiting the protein A column, these samples were not at a pH low enough to induce aggregation, this was confirmed by SEC analysis.

#### 2.3.3 Size exclusion chromatography

0.1 M sodium phosphate, 276 mM NaCl, pH 6.3 was used as the mobile phase buffer for SEC. The flow rate was 1 mL/min and the injection volume was 20 μL. Peaks were detected by absorbance at 280 nm. Before injection onto the SEC column all samples were spun at 13,000 rpm to remove any precipitated protein material.

#### 2.3.4 Data analysis

Statistical analysis and data fitting were carried out using OriginLab^®^ OriginPro 9.0 software.

## 3 Results and discussion

### 3.1 Experimental design

**Fig. 1:**
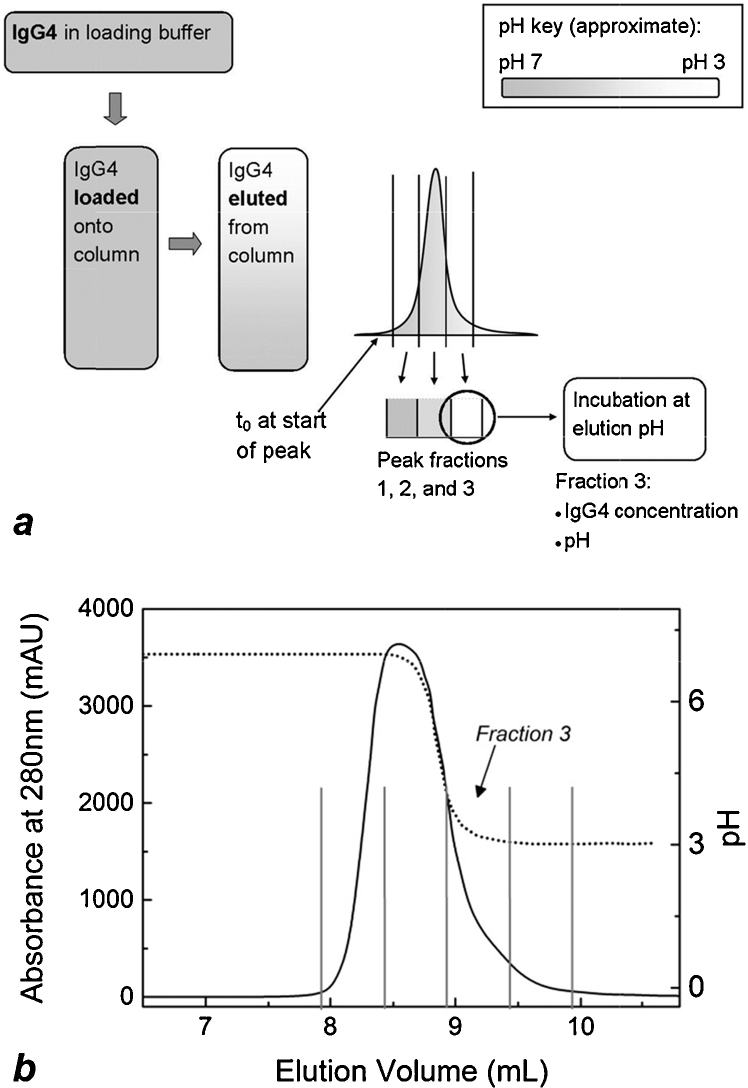
Schematic of experimental design where incubation at low pH follows protein A chromatography (a). Incubation is assumed to begin when the elution peak begins, denoted *t*_0_. Fractions containing approximately the first two thirds of the elution peak were at pH conditions considerably higher than the elution buffer pH. IgG concentration and pH in fraction 3 were the conditions reproduced in solution experiments for comparability with column experiments. A precise representation of the experimental outcome can be seen in (b), a chromatogram from a protein A chromatography run. The solid line (left *y*-axis) represents the IgG elution peak and the dotted line (right *y*-axis) indicates the pH of column effluent, as measured by the in-line pH probe. Here, flow of elution buffer begins at 7 mL elution volume. The last two fractions of the elution peak, indicated within vertical lines in (b), were at pH conditions low enough to induce aggregation.

Initial experiments were done in solution under a range of low pH conditions and IgG4 concentrations in order to determine the basic behaviour of the mAb at low pH and identify a useful pH range to work in. Typical conditions and time-courses for protein A chromatography and virus inactivation at low pH were taken into account. Time-consumption and reproducibility of experiments was also considered. Additionally, the formulation buffer of the purified mAb contributed to experimental design decisions; as the formulation buffer already contained a high concentration of glycine (0.27 M), glycine–HCl buffers were used to lower the pH in solution experiments, and for product elution in column experiments. Glycine–HCl is often used as an affinity chromatography elution buffer, though citrate or acetate buffers are more common in bioprocessing when a slightly higher elution pH is required [23–26]. In the case of this work, using glycine–HCl reduced the number of sample manipulation steps and potential confounding effects this might have on aggregation data. The pH range chosen for experiments covers a larger range than would usually be considered for industrial purification [6,7,9,10,21], as it is required to plot correlations between aggregation rates and low pH. Column experiments were carried out as shown in Fig. 1. Incubation at the elution pH was assumed to begin at the first sign of the elution peak (increase in absorbance at 280 nm), denoted *t*_0_ in Fig. 1a. It is assumed that the mAb has been exposed to the elution buffer at this stage. The column effluent does not approach pH 3 until the tail end of the peak, i.e. *Fraction* 3, but marking *t*_0_ earlier eliminates the possibility of exaggerating the column effect on aggregation rate. The IgG4 concentration in *Fraction* 3 (Fig. 1b) was within the range tested in solution, supporting comparability between in-solution and on-column data.

**Fig. 2:**
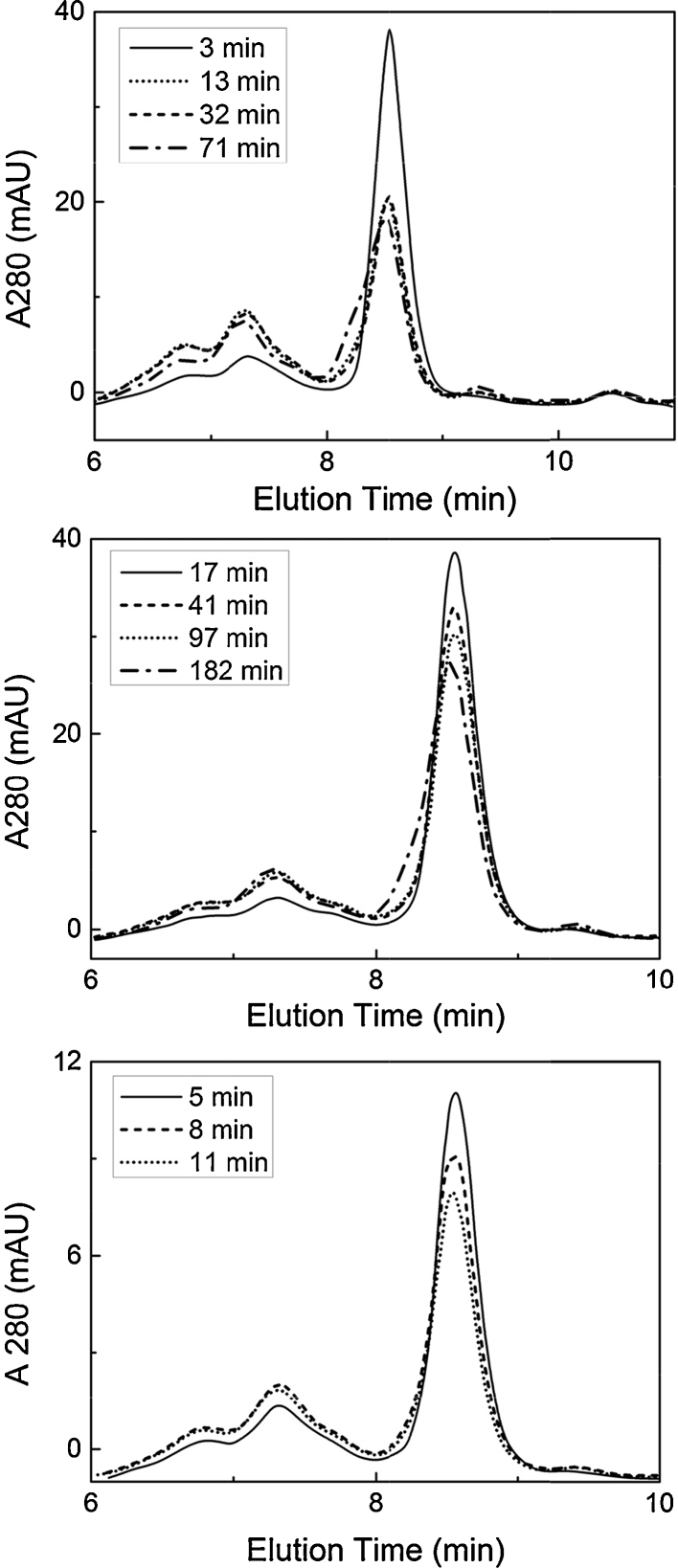
SEC chromatograms for IgG4 incubated under a range of low pH conditions. Top: pH 2.78 in solution. Middle: pH 3.05 after elution from column loaded with 12 mg IgG. Bottom: pH 2.92 after elution from column loaded with 25 mg IgG. Legends show incubation times to the nearest minute.

Size exclusion chromatography was used to quantify IgG monomer and high molecular weight (HMW) species in the test samples. Monomer loss over time was chosen as the basis for quantification of aggregation rates. Under all sufficiently harsh conditions tested, monomers were partially converted to a range of high molecular weight species over time as can be seen in Fig. 2. A few major observations were made. Firstly, regardless of (perceived) harshness of conditions, with sufficient time, incubation at low pH resulted in an increasing front shoulder to the monomer peak and a small shift of the whole peak in the direction of shorter elution time. This is indicative of a monomer either with increased molecular weight, or with altered physical character that could accelerate its transit through the SEC column [5]. Harsher conditions and/or longer incubation times instigated these changes in the monomer peak. The *pronounced* front shoulder on the monomer peak (Fig. 2, top) may represent an individual population of monomer in a molten globule state [27]. Alternatively, it may be due to an oxidised species such as the oxidised tryptophan IgG2 monomer “pre-peak” species characterised by Wong et al. [28]. Another observation was that decrease in monomer peak area was proportional to increase in HMW species peak area, up to a point. After this point, (see top and middle plots in Fig. 2), the peak area for HMW species did not increase and the monomer front shoulder developed further; in the bottom plot (Fig. 2) the monomer peak continued to decline without either significant development of the monomer front shoulder or an increase in HMW species. These results indicate that aggregates whose molecular weights exceed the exclusion limit of the SEC column may form to varying extents depending on the conditions. Such species may have been retained inside the column or HPLC system, or been removed by centrifugation prior to sample injection onto the column.

Overall, it was concluded that reduction in monomer peak area as determined by SEC provided an accurate measure of aggregation rate. In cases of long or harsh incubation, the leading shoulder developing on the monomer peak was counted as normal monomer when plotting the data; the implications of this are discussed in Section 3.3.1.

### 3.2 Rate of monomer loss at low pH in solution

**Fig. 3:**
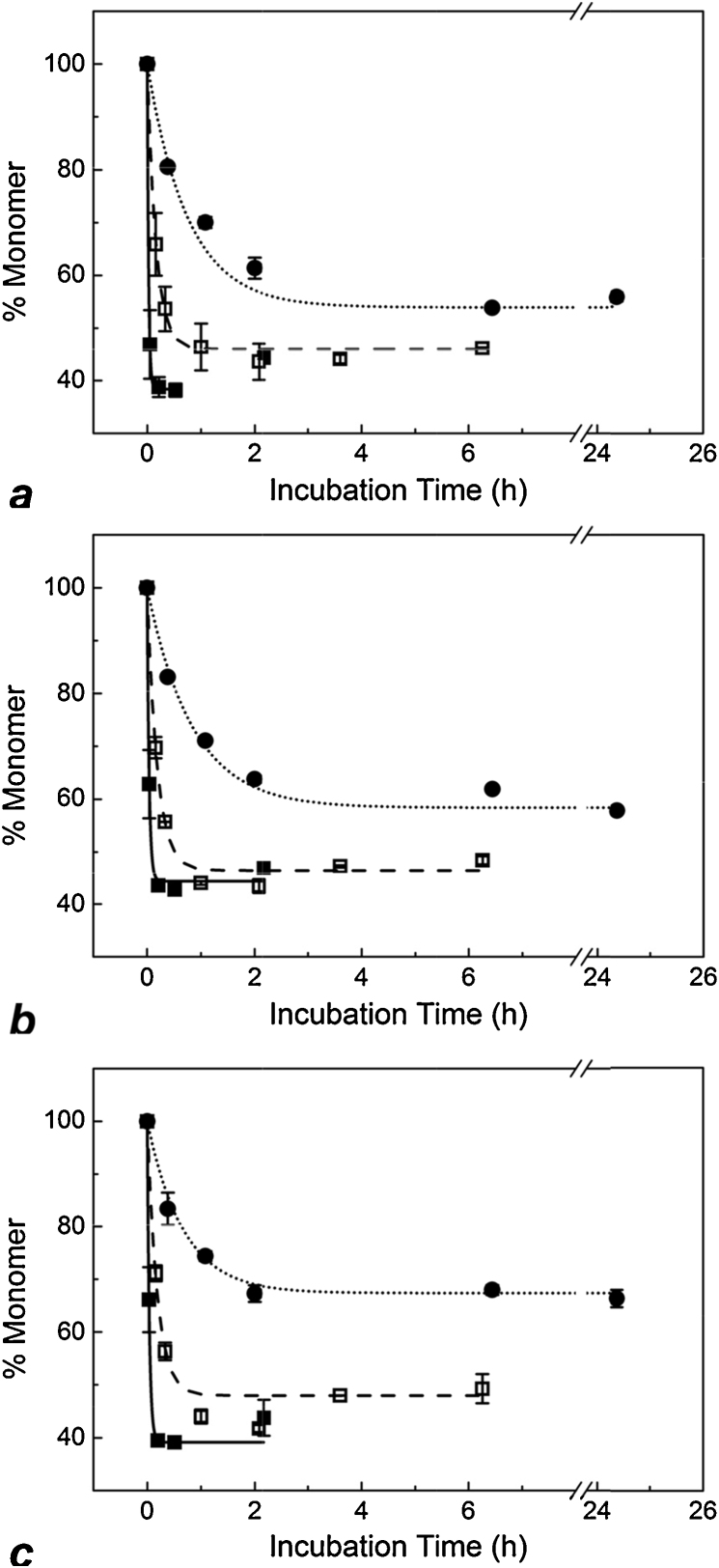
IgG monomer loss over time in solution at (a) 0.9 mg/mL, (b) 2.7 mg/mL and (c) 4.5 mg/mL. Different symbols represent different incubation pH conditions: circles, pH 3.03; open squares, pH 2.95; filled squares, pH 2.78. Error bars show the standard deviation for each point based on full experimental repeats, *n* = 2. Exponential decay curves were fitted to the data using the equation *y* = *y*_0_ + *Ae*^*R*_0_.*x*^ (see Section 3.2 for equation specifics). In (a), for pH 2.78 the last time point (2.18 h) was excluded from the curve fit (see Section 3.3.1). All fits were significant with adjusted *r*^2^ > 0.99 and *P* < 0.01.

The IgG4 was found to be highly sensitive to pH within a critical range. At pH conditions lower than pH 2.8 aggregation was rapid; plateau occurred in less than 30 min. However, at pH > 3.0 completion took approximately 4 h. It should be noted that the plateau was not found at the point of complete monomer loss, and varied depending on pH conditions; lower pH conditions resulted in greater total monomer loss (Fig. 3). It is possible that what appears as a plateau in monomer depletion actually represents a point of reversible equilibrium between species at one or more stages of the aggregation process. An ancillary experiment was carried out in which monomer purified from initial aggregation runs was re-exposed to low pH under the same conditions as in the initial runs. Recovered monomer was found to display near-identical aggregation behaviour to initial monomer (data not shown). This indicates that the surviving monomer population is not distinct from the aggregating portion, supporting the possibility of an equilibrium mechanism. Despite the fairly large IgG concentration range tested (five-fold), concentration appeared to have little effect on aggregation kinetics, and observed differences in plateau did not follow a clear trend. Thus we hypothesise that aggregation plateau was determined by pH-dependent equilibrium between unfolded and native or re-folded monomers. While this could provide an interesting topic for more detailed investigation, it will not be the focus of this study. Instead, we concentrate primarily on initial aggregation *rates* as well as apparent total monomer loss.

All data sets were fitted with exponential decay curves using the following equation:

(2) $$y=y_{0}+A.{\text{e}}^{-R_{0}.x}$$

where *y*_0_ is the plateau point (total monomer remaining), *A* is the initial value (total monomer lost), *R*_0_ is the rate constant (h^−1^) and *x* is incubation time (h).

**Fig. 4:**
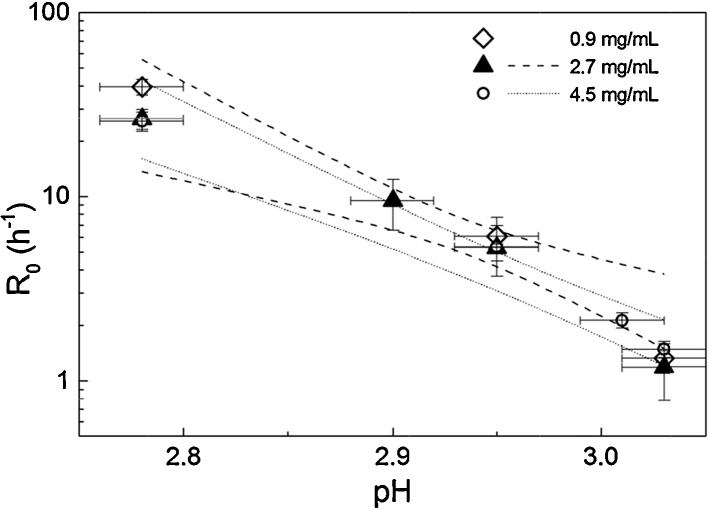
Semi-log plot of monomer decay rate, *R*_0_, against pH in solution at three different IgG concentrations. *Y*-error bars show the standard error for the *R*_0_ parameter (Eq. (2)) obtained from least squares fitting; *x*-error bars represent pH measurement error. Linear regression was applied for concentrations 2.7 mg/mL and 4.5 mg/mL; for clarity, 95% confidence intervals only are shown for these fits. A trend has not been fitted to the 0.9 mg/mL data because there are few data points.

The value of interest resulting from fitting curves to the data is the rate constant, *R*_0_. The rate constant provides a convenient comparator for chromatography experiments, similarly to that used by Shukla et al. [9]. Rate constants varied most with pH, while IgG4 concentration had a limited effect on *R*_0_ values, within the concentration range tested, as seen in Fig. 4. To verify concentration effect, or lack thereof, *R*_0_ was plotted against pH for the three IgG concentrations tested and linear regression with 95% confidence limits was applied to each data set. There were few data points at 0.9 mg/mL so this data set was not fitted. Confidence intervals of the linear fits for 2.7 mg/mL and 4.5 mg/mL overlapped, indicating that IgG concentration did not significantly affect *R*_0_ in solution (Fig. 4). This is useful in terms of allowing some flexibility in IgG concentration for column experiments, as elution fractions may not be of precisely the same concentration for every run.

#### 3.2.1 Negligible effect of maltose

Maltose has been known to stabilise proteins and reduce aggregate formation [17]. The IgG4 initial formulation contained 1% maltose; subsequently, low concentrations of maltose were present in solution experiments (0.05–0.25%). Maltose was not added to elution buffers for column experiments so was not present during those incubations. Thus it was necessary to confirm that maltose was not reducing aggregation rates in solution. Additional solution experiments were carried out at pH 2.90 with maltose at concentrations from 0.15% to 4.0%, and 2.7 mg/mL IgG4. Maltose had no significant effect on IgG aggregation rate in these experiments (data not shown). Low maltose concentrations (0.25–1.5%) also had no discernible effect in solution from 0.9 to 4.5 mg/mL IgG4, pH 3.0 (data not shown). Subsequently, maltose was not added to protein A elution buffers.

### 3.3 Effect of column step on rate of monomer loss at low pH

**Fig. 5:**
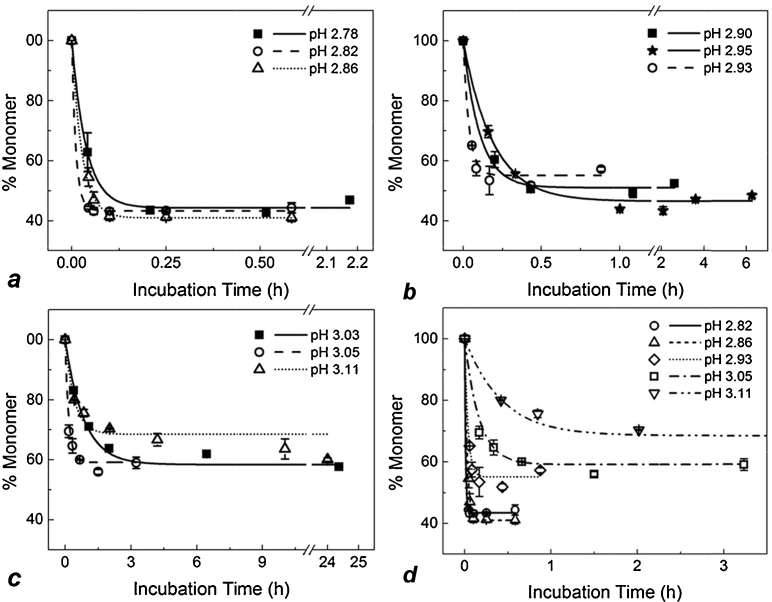
Rates of monomer loss at 2.7 mg/mL IgG under different pH conditions in solution only (filled symbols and solid lines) and after elution from a protein A chromatography column (open symbols, dashed lines and dotted lines) (a–c). Different pH conditions are shown in the plot legends. Curves for column runs across the full pH range are shown together in (d). Error bars show the standard deviation for each time point based on full experimental repeats, *n* = 2. All data sets were fitted with exponential decay curves as was done for initial solution only data. All fits were significant with adjusted *r*^2^ > 0.98 and *P* < 0.01.

In Fig. 5a–c monomer loss over time under various pH conditions is plotted for column and solution experiments side by side. All column experiment data was fitted with exponential decay curves as was done for solution data. Fig. 5d shows all column experiment curves side by side on a single unbroken scale. Examining the plots in Fig. 5, the initial rate of monomer consumption and the position and behaviour of the plateau can be compared for the different test conditions. Due to the sharp drop-off in pH sensitivity between pH 2.7 and pH 3.0, the data in Fig. 5a–c is displayed in three narrow ranges: pH 2.78–2.86 (a), pH 2.90–2.95 (b) and pH 3.03–3.11 (c). Note that the *x*-axis scales differ for each plot and also contain scale breaks. The pH values indicate the pH at which the IgG was incubated after elution from the column, which was the same as the pH of the original elution buffer. In all cases, inclusion of the protein A chromatography step immediately before incubation resulted in a faster rate of monomer decay at any given pH. Exponential decay kinetics to a point of apparent equilibrium was maintained in column experiments as in solution. This suggests that the chromatography step does not significantly change the basic aggregation mechanism, but does accelerate it.

#### 3.3.1 Monomer leading shoulder

As can be seen largely in Fig. 3 and Fig. 5, though a general plateau was reached in all experiments, it was not completely stable. In some cases (typically the harsher conditions) monomer concentration appeared to drift up again. This may be explained by the apparent formation of an altered monomer species, as seen in Fig. 2. The (extra) area contributed by this peak shoulder was counted as normal monomer in “% *Monomer*” calculations (see Section 2.3.1). Excluding the shoulder area would result in stabilisation of % *Monomer* values at the plateau, rather than upward drift up. On the other hand, whether the monomer shoulder species should be considered a precursor to the aggregated form, or even one of its break-down products, is contestable [27]. Thus, data points that drift up after the plateau were counted as “normal” for the purpose of curve fitting *unless* the fit was rejected by the software (see Fig. 3a, pH 2.78). Nonetheless, it is worth noting that the shoulder may represent a degraded form of the product whose bioactivity differs from that of the native monomer [28]; this could provide an interesting subject for further investigation. In other cases, after a period of stability monomer concentration began to drop (Fig. 5c), this is discussed below.

In column experiments the shoulder developed to a similar extent but more quickly than in solution experiments, supporting the model of acceleration of the mechanism of aggregation when the chromatography step was present.

#### 3.3.2 Variation in monomer plateau

Within the lowest pH range, pH 2.78–2.86, the monomer plateau consistently fell between 40% and 45% of the initial concentration for both solution and column experiments (Fig. 5a and d). Within the highest pH range, pH 3.03–3.11, plateau points were similar for near-matching pH conditions (e.g. solution pH 3.03 versus column pH 3.05). The trend of reduced total monomer loss with increasing pH was matched by the column experiment data at pH 3.11, all in all the highest pH tested, with the plateau being at the highest percentage monomer compared to all other test conditions. For the pH 3.11 column experiment a slow linear drop in monomer continues between 4 h and 24 h, meaning that exponential decay may not be the best fit available for the data in this region and a different mechanism of aggregation may prevail. For the purpose of this study, an exponential decay fit was used as it described the initial period of rapid monomer loss well, giving an adjusted *r*^2^ value of 0.985 with significance *P* < 0.01 for pH 3.11 (Fig. 5). It is possible that above a certain pH, or even after a certain duration, the system transitions to a different kinetic mechanism.

In other cases upward drift in the plateau occurred, but this can largely be attributed to the presence of monomer leading shoulder species described above.

**Fig. 6:**
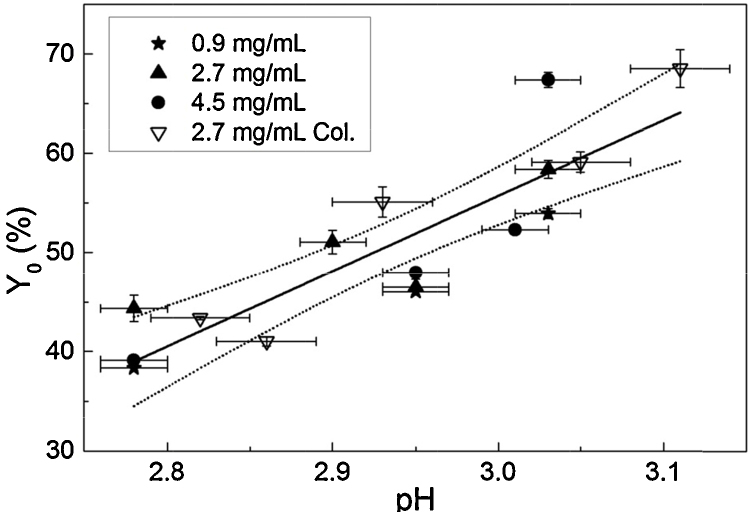
*Y*_0_ (plateau point parameter) from exponential decay curve fits plotted against pH at 0.9 mg/mL, 2.7 mg/mL and 4.5 mg/mL in solution, and at approximately 2.7 mg/mL after protein A chromatography. *Y*-error bars show the standard error for the *Y*_0_ parameter (Eq. (2)) obtained from least squares fitting; *x*-error bars represent pH measurement error. The data was pooled and fitted with a linear trend (solid line); error weighting was not used for the fitting. The 95% confidence interval is shown as dotted lines. The fit was significant with an adjusted *r*^2^ of 0.75 and *P* < 0.01.

In summary, the plateau point was not considered a robust parameter to quantify the aggregation that occurred under the given conditions. This is further illustrated in Fig. 6, where the relationship between pH, *y*_0_ values and IgG concentration for solution-only and column experiments is displayed. The general trend of decreasing monomer loss with increasing pH can be seen, and it is also apparent that the effects of IgG concentration and the column step on *y*_0_ are not well defined.

#### 3.3.3 Influence of column step on *R*_0_

Focussing on the initial period of rapid monomer loss under the various conditions displayed in Fig. 5, it can be seen that the column step had a considerable effect on the rate at which monomers were converted to aggregate species during the low pH hold. Looking at Fig. 5b, between pH 2.90 and pH 2.95 it is clear that that rate of monomer loss is fastest for the column experiment. Comparing pH 3.03 solution data to pH 3.11 column data (Fig. 5c), the initial decline in monomer progresses at a similar rate for both experiments; this is significant considering the extreme pH sensitivity of the system.

Fig. 5 allows the kinetics of individual cases to be assessed and compared. Due to the pH sensitive nature of the experiments, direct comparison of column and solution data is difficult because of small differences in pH. Individual fitted parameters, *R*_0_ and *y*_0_, were plotted against pH to more clearly delineate the effect of the chromatography step.

**Fig. 7:**
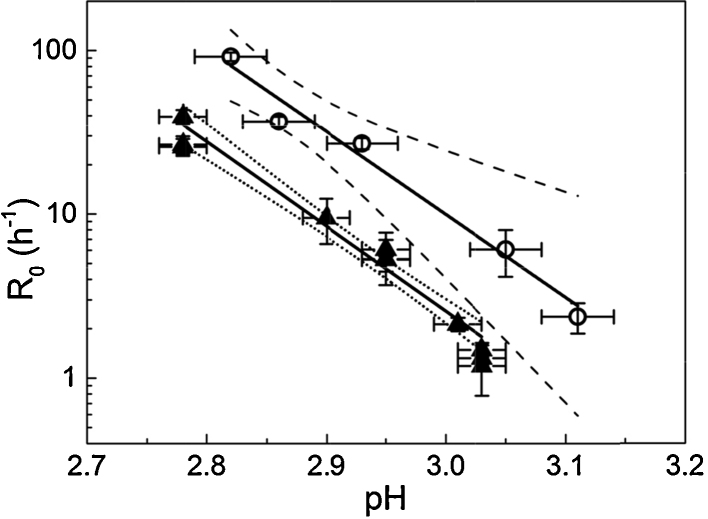
Semi-log plot of monomer decay rate, *R*_0_, against pH for solution-only experiments (filled triangles) and experiments including a protein A chromatography step (open circles). For column experiments, the typical concentration of the incubated elution fraction was 2.7 mg/mL. Solution-only data seen in Fig. 4 was pooled due to overlapping 95% confidence intervals for the linear trends. For solution-only data points, *x* error bars represent the measurement error of the laboratory pH probe/metre, as reported by the manufacturer. For chromatography experiments, *x* error bars correspond to measured error based on three replicate measurements (with a micro pH probe). For both solution-only and column experiments, y error bars represent the standard error for the *R*_0_ parameter (Eq. (2)) obtained from least squares fitting. A linear trend was fitted to each data set, as shown by solid lines; dashed/dotted lines represent 95% confidence limits for each fit. Both fits were significant with *P* < 0.01 and adjusted *r*^2^ of 0.97 and 0.90 for solution-only and column experiments, respectively.

Fig. 7 illustrates how the protein A chromatography step causes a shift in the rate of monomer loss compared to low pH incubation alone. It is clear that two separate trends exist for *R*_0_ against pH for solution-only and on-column experiments. In Fig. 7, 95% confidence bands were calculated using the standard error for the *R*_0_ parameter (obtained from least squares fitting) for weighting. The linear fit lines for column and solution-only *R*_0_ values are almost parallel; this supports the theory that the aggregation mechanism is accelerated, but not significantly altered, by the chromatography step. The data represents the minimum difference between after-column and solution-only rates, in that the earliest reasonable start time for low pH incubation was used for column experiments (see Section 3.1).

It should be noted that the *y* axis in Fig. 7 is scaled logarithmically; what appears as a linear fit is actually exponential. This is of interest as it shows how the data collected is centred on a tipping point for aggregation behaviour of the IgG4; two orders of magnitude in aggregation rate are covered in approximately 0.3 pH units. Outside the range of conditions tested aggregation becomes either unacceptably rapid, or slow to the point of practical insignificance (in the context of an early/mid purification process). It is important to consider that the column step has an effect despite the narrow range in which it was possible to quantify effects using the methods described. In effect, the column step seems to push the tipping point in the direction of milder conditions. A related result was seen by Gagnon et al. for an IgG1. They found that adjusting to pH 3.0 after elution from protein A (at pH 3.5) resulted in considerable aggregate formation which was not observed in the absence of the chromatography step. It was concluded that elution from protein A increased the protein's “vulnerability” to a secondary stress, such as pH [13].

## 4 Conclusion

The data we have gathered captures the behaviour of an IgG4 within a fairly compact experimental space. The IgG4 demonstrates highly pH-dependent and apparently concentration-independent aggregation behaviour. Given these observations, we hypothesise that the aggregation behaviour is governed predominantly by a pH-dependent unfolding/re-folding equilibrium. Our data shows that the chromatography step causes aggregation rates to increase, while other aspects of the aggregation kinetics appear largely un-affected. One possible molecular mechanism is that the conformational change, as well as changes in the hydration layer around the protein, required for protein adsorption and subsequent desorption, alter the structure of the IgG, possibly exposing regions of the protein involved in unfolding transitions. Being in this condition, immediate exposure to the acidic environment could increase the possibility of the protein unfolding and losing native structure to some extent before progression to aggregate formation during the pH hold [13,27,29].

It should also be considered that only the tail end of the protein A elution peak was assessed in this work, as all prior effluent was at a pH too high to induce aggregation. The peak tail may represent a monomer sub-population with a higher affinity for the ligand. It is possible that these monomers bind more-tightly than early-eluting species, undergo increased structural perturbation during adsorption and desorption, and are thus more likely to aggregate, increasing the rate. This type of phenomenon, whereby a strongly bound sub-population was generated, has been described previously and investigated in detail for hydrophobic interaction chromatography [30,31]. Heterogeneity in recombinant protein products is commonly documented, with species raging from distinct charge variants to product molecules with a single erroneous amino acid substitution [32]. A future extension of this work could investigate heterogeneity across the elution peak, and subsequent effects on aggregation behaviour.
